# Supplementary material for: Real-World Efficacy and Safety of Apixaban vs. Warfarin in Obese Atrial Fibrillation Patients: Propensity Matching Analysis
Source: Biomedicines. 2025 Feb 17;13(2):490. doi: 10.3390/biomedicines13020490 (PMC11853457; doi:10.3390/biomedicines13020490)
Supplement: Supplementary file 1 [file biomedicines-13-00490-s001.zip › biomedicines-3394722-supplementary.pdf]

Supplementary materials

Supplementary Figure S1. The study events presented periodically (3-months, 6-months, 1-year, 2-years). **(A)** Mortality. **(B)** Stroke. **(C)** Venous thromboembolism (VTE). **(D)** Bleeding.

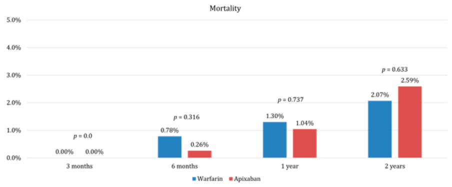

(A)

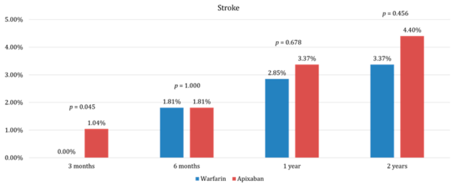

(B)

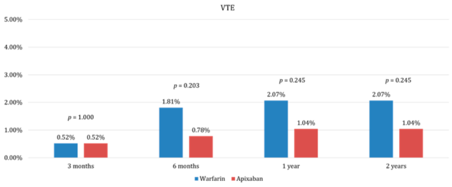

(C)

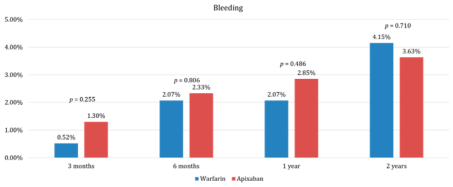

(D)
